# Outbreak of pandemic influenza A/H1N1 2009 in Nepal

**DOI:** 10.1186/1743-422X-8-133

**Published:** 2011-03-23

**Authors:** Bal Ram Adhikari, Geeta Shakya, Bishnu Prasad Upadhyay, Khagendra Prakash KC, Sirjana Devi Shrestha, Guna Raj Dhungana

**Affiliations:** 1 Department of Health Services, Molecular Virology Research Division, National Public Health Laboratory, Kathmandu, Nepal; 2 Department of Health Services, Department of Microbiology, National Public Health Laboratory, Kathmandu, Nepal

## Abstract

**Background:**

The 2009 flu pandemic is a global outbreak of a new strain of H1N1 influenza virus. Pandemic influenza A (H1N1) 2009 has posed a serious public health challenge world-wide. Nepal has started Laboratory diagnosis of Pandemic influenza A/H1N1 from mid June 2009 though active screening of febrile travellers with respiratory symptoms was started from April 27, 2009.

**Results:**

Out of 609 collected samples, 302 (49.6%) were Universal Influenza A positive. Among the influenza A positive samples, 172(28.3%) were positive for Pandemic influenza A/H1N1 and 130 (21.3%) were Seasonal influenza A. Most of the pandemic cases (53%) were found among young people with ≤ 20 years. Case Fatality Ratio for Pandemic influenza A/H1N1 in Nepal was 1.74%. Upon Molecular characterization, all the isolated pandemic influenza A/H1N1 2009 virus found in Nepal were antigenically and genetically related to the novel influenza A/CALIFORNIA/07/2009-LIKE (H1N1)v type.

**Conclusion:**

The Pandemic 2009 influenza virus found in Nepal were antigenically and genetically related to the novel A/CALIFORNIA/07/2009-LIKE (H1N1)v type.

## Background

The 2009 flu pandemic is a global outbreak of a new strain of H1N1 influenza virus, often referred to as swine flu [1]. The virus is a novel strain of influenza [2]. This new pandemic H1N1 influenza strain contained genes from five different flu viruses: North American swine influenza, North American avian influenza, human influenza, and two swine influenza viruses typically found in Asia and Europe [3]. Due to the genetic mutations in its hemagglutinin (HA) protein, the influenza viruses can escape from the host defense mechanisms and thus to be able to continuously infect human and other species [4,5].

On June 11, 2009, the ongoing outbreak of Influenza A/H1N1 was officially declared by the WHO to be the first influenza pandemic of the 21^st ^century with new strain of Influenza A virus subtype H1N1 identified in April 2009 [6]^. ^Till May 30, 2010 worldwide update by World Health Organization (WHO) more than 214 countries have reported laboratory confirmed cases of pandemic influenza H1N1 2009, including over 1,8,114 deaths [7].

Nepal has started screening febrile travelers with respiratory symptoms from affected countries for the Pandemic influenza A (H1N1) since April 27, 2009, and the first case was detected on June 21, 2009 and introduction of the disease to the country was declared on June 29. Community transmission of Pandemic Influenza A/H1N1 2009 was declared on 15 October onwards.

This study reflects the actual outbreak situation and its severity of Pandemic Influenza A/H1N1 in Nepal.

## Methods

This is a Laboratory based prospective cross-sectional study carried out at National Public Health Laboratory from April 2009 to May 2010. Initially during the pandemic declaration of H1N1, samples were collected from patients of all age groups from both gender meeting the criteria of case definition of influenza like illness (ILI) with history of international travel from country of confirmed Pandemic H1N1 or close contact with confirmed H1N1 infected persons or with shortness of breathing or hospital admitted patients. As the community outbreak of pandemic influenza A/H1N1 was declared, sample collection was done by random sampling method from the patients meeting case definition of ILI. The patients already on antiviral treatment were excluded from the study. Influenza like illness (ILI) is defined as those who has fever ≥ 38°C with at least one respiratory symptoms such as cough, rhinorrhoea or sore throat [8].

Posterior pharyngeal swabs were collected into Viral Transport Medium (VTM). For transportation from outside hospitals and outbreak area, Specimens were kept on ice box (2-8°C) with bio-safety precaution and transported to the National Public Health Laboratory within 48 h after collection.

At NPHL, Laboratory diagnosis of Pandemic influenza A/H1N1 was made by one step probe-based Real Time-Polymerase Chain Reaction (PCR) on Posterior pharyngeal swabs. Initially, samples were tested for Universal influenza A, Once Universal influenza A was positive, it was further tested for Swine specific A followed by Swine specific H1. According to CDC guideline, Presumptive positive for Pandemic influenza A/H1N1 was declared if RT-PCR give positive results from either swine A or swine/H1 or positive from both tests [9]. All the specimens which give presumptive negative for Pandemic influenza A/H1N1 but universal influenza A positive were tested for seasonal influenza A (H1, H3 and H5) and those specimens negative for all swine A/swine/H1 and universal influenza A were tested for Influenza B.

### Quality assurance and accreditation of Molecular Laboratory

five samples presumptive positive for Pandemic influenza A/H1N1 at our laboratory were retested at Microbiology Department of University of Hongkong, Hongkong and the concurrence was 100%. We are regularly participating in the WHO External Quality Assessment Programme for the detection of Influenza virus type A and type B by PCR from panel 8.

### RT-PCR Technique

RNA was extracted from 140 μl aliquot of posterior pharyngeal swabs in Viral Transport Medium (VTM) by using viral RNA extraction kit (QIAmp, Qiagen) [10]. 5 μl of RNA extract was mixed with 5.5 μl of Nuclease free water, 0.5 μl of forward primer, 0.5 μl of Reverse primer, 0.5 μl of Probe, 0.5 μl of Superscript™III RT/Platinum *Taq *mix, 12.5 μl of 2× PCR Master Mix making total reaction volume 25 μl.

Three separate primer/probe sets for Universal influenza A(Inf A), Universal swine specific (swFluA) and Swine specific H1 (swH1) were used. The internal positive control for human nucleic acid was RNase P primer/probe set which targets for human RNase P gene. The primers and probes used in the RT-PCR system were as described in Table 1.

**Table 1:** Primers and Probe sets used for the diagnosis of Pandemic Influenza A/H1N1,

*Primers and Probes*	*Sequence (5' > 3^'^)*	*Working Concentration*
*Inf A Forward*	*GAC CRA TCC TGT CAC CTC TGA C*	*40 μM*
*Inf A Reverse*	*AGG GCA TTY TGG ACA AAK CGT CTA*	*40 μM*
*Inf A Probe*	*TGC AGT CCT CGC TCA CTG GGC ACG*	*10 μM*
*SW Inf A Forward*	*GCA CGG TCA GCA CTT ATY CTR AG*	*40 μM*
*SW Inf A Reverse*	*GTG RGC TGG GTT TTC ATT TGG TC*	*40 μM*
*SW Inf A Probe*	*CYA CTG CAA GCC CA "T" ACA CAC AAG CAG GCA*	*10 μM*
*SW H1 Forward*	*GTG CTA TAA ACA CCA GCC TYC CA*	*40 μM*
*SW H1 Reverse*	*CGG GAT ATT CCT TAA TCC TGT RGC*	*40 μM*
*SW H1 Probe*	*CA GAA TAT ACA "T"CC RGT CAC AAT TGG ARA A*	*10 μM*
*RnaseP Forward*	*AGA TTT GGA CCT GCG AGC G*	*40 μM*
*RnaseP Reverse*	*GAG CGG CTG TCT CCA CAA GT*	*40 μM*
*RnaseP Probe*	*TTC TGA CCT GAA GGC TCT GCG CG*	*10 μM*

RT-PCR system was performed as per protocol provided by Center for Disease Control, USA. The following RT-PCR program was used in this study: Reverse Transcription was at 50 °C for 30 min, Taq inhibitor activation at 95 °C for 2 min, PCR amplification cycle was at 95 °C for 15 sec and 55 °C for 30 sec with 45 times repetition. Fluorescence data (FAM) was collected during the 55 °C incubation step [9].

### Analysis in RT-PCR

When all controls i.e. Negative, Positive and human Rnase P meet the requirements, a specimen is considered positive if the reaction growth curves cross the threshold line within 40 cycles. Similarly, a specimen is considered negative if growth curves do not cross the threshold within 40 cycles [9].

### Virus typing

Blindly selected few positive samples of Pandemic influenza A/H1N1 from RT-PCR were sent to the WHO reference laboratory of Center for Disease Control and Prevention Division (CDC), Atlanta. All samples were isolated and antigenically characterized by Haemagglutination Inhibition Assay (HAI).

### Statistical tools

Statistical tools like Probability were calculated by using Statistical Package for the Social Sciences (SPSS) programme.

## Results

A total of 609 patients with suspected Pandemic influenza A/H1N1 were tested at National public health laboratory during the study period. All the samples were confirmed by Real-Time PCR. Out of these samples, 172 (28.3%) were Pandemic influenza A/H1N1 positive and 130 (21.34%) cases were seasonal influenza A as in Table 2. Due to heavy work load and priority for identification of pandemic H1N1, all the Seasonal influenza-A positive cases were not sub-typed. A sub-set of 88 seasonal-A positive samples, till November 2009 were subtyped and found 70(79.5%) were seasonal H3, 8(9.09%) were seasonal H1 and 10(11.36%) were unsubtypeable seasonal influenza A. Among 219 negative cases for Universal Influenza A were tested for Influenza B and found only one positive case.

**Table 2:** Total cases of Suspected Pandemic influenza A/H1N1 (n = 609)

*Cases*	*Universal Influenza A*	*Pandemic Influenza A/H1N1*	*Seasonal Influenza A*
*Positive*	*302(49.6%)*	*172(28.3%)*	*130(21.34%)*
*Negative*	*307(50.4%)*	*437(71.77%)*	*479(78.66%)*

**Figure 1:**
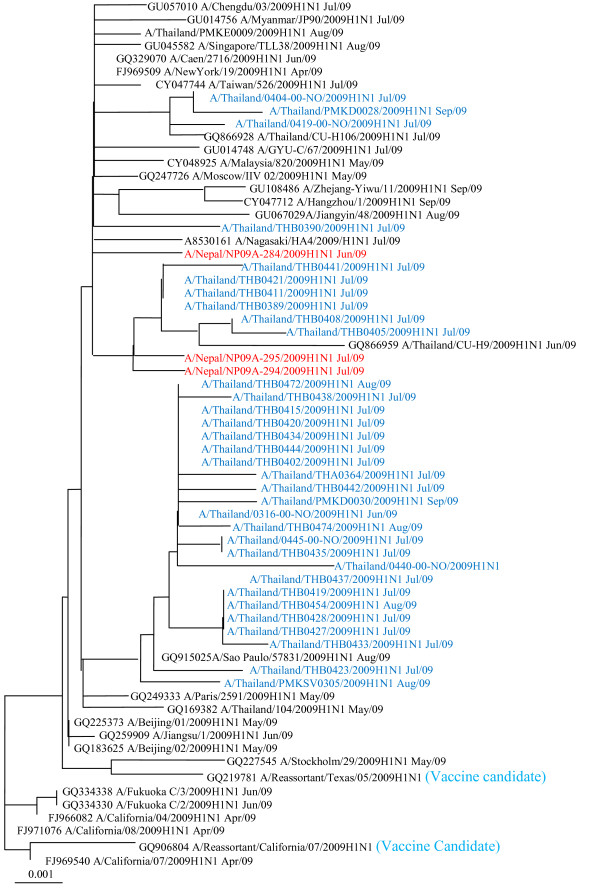
**The neighbor joining tree of influenza A/H1N1 (Swine) 2009 complete HA gene (1,701 bp)**.

Ten randomly selected positive cases of Pandemic influenza A/H1N1 by RT-PCR were isolated and antigenically characterized by Haemagglutination Inhibition (HAI) Assay in WHO reference lab US CDC in October 2009. All the characterized virus found in Nepal were antigenically and genetically related to the novel influenza A/CALIFORNIA/07/2009-LIKE (H1N1)v type (Figure 1).

**Figure 2:**
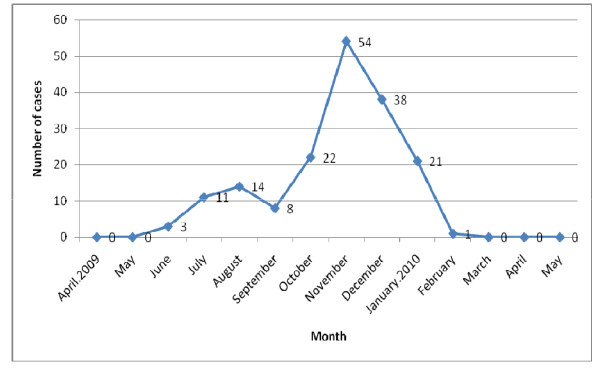
**Epidemic curve of Confirmed cases of Pandemic influenza A/H1N1 2009 (n = 172)**.

First case of Pandemic influenza A/H1N1 was detected in June 2009. Starting from the June, cases were increasing up to peak of the Pandemic Influenza outbreak in November (Figure 2**)**.

Till May 2010, total number of confirmed positive cases of Pandemic influenza A/H1N1 was 172. Out of them, 36 cases were recorded before declaration of Community transmission among 29 Nepalese citizens residing within the country or from abroad, 2 foreigner, 5 close relatives of confirmed positive cases and remaining 136 cases were found after community transmission which was declared on October 15 2009.

**Figure 3:**
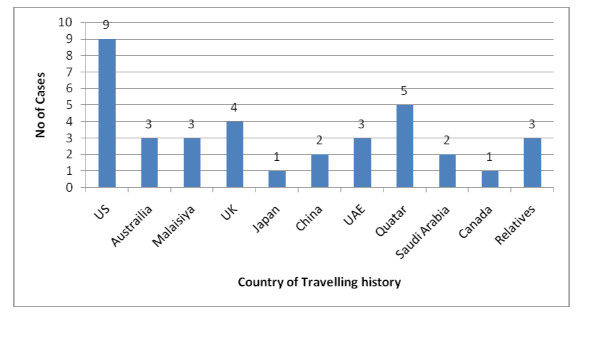
**Travel history of confirmed Pandemic influenza A/H1N1cases before community transmission (n = 36)**.

Travelling history of pandemic influenza cases before community transmission was as shown in Figure 3. Among them, largest no of cases were found from US.

**Figure 4:**
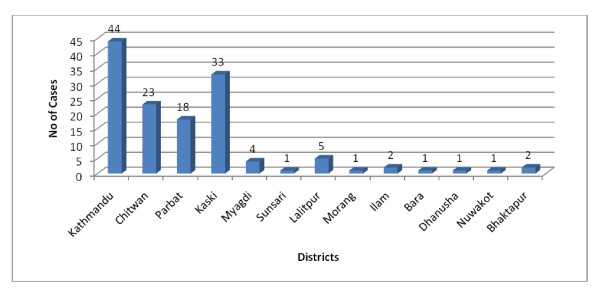
**District wise distribution of total Pandemic influenza A/H1N1 cases after community transmission (n = 136)**.

After community outbreak, most of the cases of Pandemic influenza were from Kathmandu district followed by Kaski and Chitwan (Figure 4).

**Figure 5:**
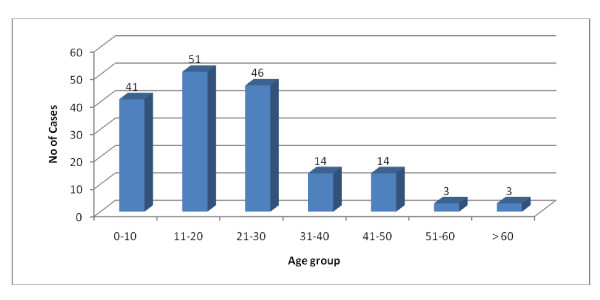
**Age wise distribution of confirmed Pandemic influenza A/H1N1 cases (n = 172)**.

**Figure 6:**
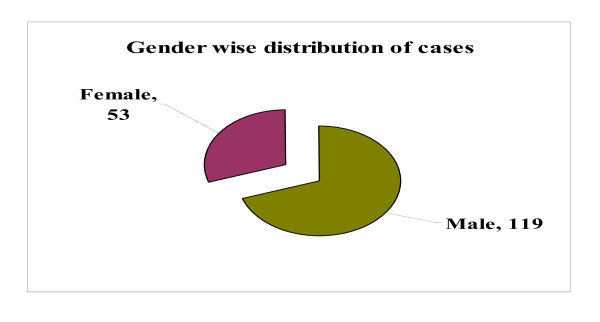
**Sex wise distribution of confirmed Pandemic influenza A/H1N1 cases (n = 172)**.

All the confirmed cases of Pandemic influenza A/H1N1 were in the range of age group from 1-74 with mean age 21 years. Most of the cases were found in the age group 11-20 followed by 21-30 and 0-10. Among the positive cases 119(69.18%) were male and 28(30.82%) were female (Figure 5 and 6).

Till May 2010, three death cases were reported due to Pandemic influenza A/H1N1. All were

**Figure 7:**
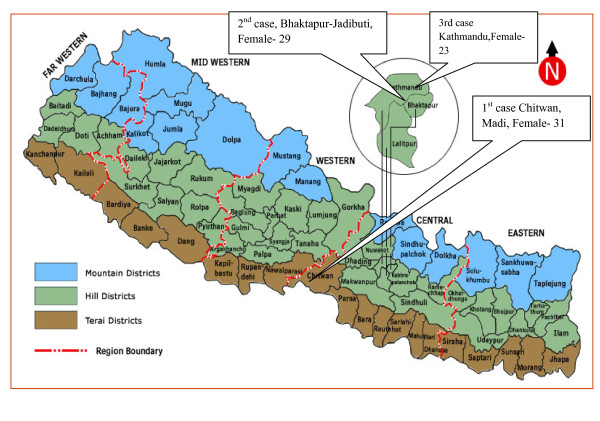
**Information of death cases in Nepal by Pandemic influenza A/H1N1**.

Female patients with age 31, 29 and 23 (Figure 7).

## Discussion

Since the middle of March 2009, infections with the new influenza A (H1N1) strain started to occur in Mexico, and the first two cases in the United States occurred in late March 2009, although they were not confirmed until April 15, 2009 [11]. The rapid global spread indicates towards the influence of international air travel on influenza [12]. According to the data from WHO till March 2010, this new influenza A (H1N1) was estimated to have a case-fatality rate (CFR) of 1.28%.

In this study, by May 28 2010, a total of 609 suspected patients were tested for Pandemic influenza A/H1N1 2009. Out of the collected samples, 130 (21.34%) were seasonal influenza A positive and 172 (28.3%) were Pandemic influenza A/H1N1 positive. The mean age of patients with confirmed Pandemic influenza A/H1N1 was 21, whereas the mean age of the positive cases of Seasonal influenza A was 28. Patients with pandemic (H1N1) 2009 were significantly younger (p = 0.04, χ2 = 8.21) than patients with Seasonal influenza A (< 20 years).

A characteristic feature of the H1N1 pandemic is that it disproportionately affected so far children and young adults [13]. Similar results were observed in different parts of the world: In Saudi Arabia, the age of the cases ranged between 1 and 56 years with mean (SD) of 24.2 (14.4) years [14]. One of the early studies from the USA showed that although the age of pandemic influenza A (H1N1) patients in the study ranged from 3 months to 81 years, 60% of patients were 18 years of age or younger. In most countries, the majority of Pandemic influenza A (H1N1) cases have been occurring in young people, with the median age estimated to be 12 to 17 years in Canada, the USA, Chile, Japan, and the UK. Most of the pandemic influenza cases in young age people indicate towards partial immunity to the virus in the older population [15].

The overall case fatality ratio (CFR) found in this study was 1.74% as 3 death cases were reported till May 28, 2010. All the death cases were found in female with below 35 years of age. The data found in this study is similar with the other literatures. The overall case fatality rate has been less than 0.5%, and the wide range of estimates (0.0004 to 1.47%) reflects uncertainty regarding case ascertainment and the number of infections [16-18]. The case fatality rate for symptomatic illness was estimated to be 0.048% in the United States [19] and 0.026% in the United Kingdom [20]. In contrast to seasonal influenza, most of the serious illnesses caused by the pandemic virus have occurred among children and nonelderly adults, and approximately 90% of deaths have occurred in those under 65 years of age [21].

## Conclusion

In Nepal, mostly young aged people were affected by the wave of this pandemic influenza and all the isolated pandemic influenza A/H1N1 2009 virus were antigenically and genetically related to the novel A/CALIFORNIA/07/2009-LIKE (H1N1)v type.

## Competing interests

We have no competing interest to declare. This study is the property of National Public Health Laboratory though World Health Organization and Center for Disease Control and Prevention (CDC) supported by providing Primers/probes, reagents and kits for the study.

## Authors' contributions

BA has made great contribution to design, acquisition, interpretation and analysis of data and overall frame out of this article. He has actively involved for the optimization of Real-Time PCR during beginning of this study. His contribution during the analysis of Real-Time PCR results made easy for drafting the article. GS has been involved for drafting the manuscript and given final approval for the publication. BU has actively participated in the laboratory work and co-ordinate overall activity of molecular diagnosis. KPK help for the sample management during Pandemic H1N1 outbreak and suggests some minor mistakes during preparation of this manuscript. SDS helped for the sample management during Pandemic H1N1 outbreak. She has contributed by proof reading of this manuscript. GRD has contributed in diagnostic part of this study. All authors read and approved the final manuscript.

## Author's information

Bal Ram Adhikari is working as Microbiologist in Virology Research Unit of National Public Health Laboratory. His interest is in Molecular as well as epidemiology based Research. He has completed Masters Degree in Medical Microbiology from Tribhuvan University. Dr Geeta Shakya is Director of National Public Health Laboratory and has done MD in Pathology and Immunology. Bishnu Prasad Upadhyay is Senior Medical Technologist in Virology Research Division of National Public Health Laboratory and has completed Masters Degree in Tropical Medicine from Mahidole University. Khagendra Prakash KC is working as Microbiologist in Virology Research Division of National Public Health Laboratory. Sirjana Devi Shrestha is working as Microbiologist in Microbiology Department of National Public Health Laboratory

Guna Raj Dhungana is working as Medical Technologist in Virology Research Division of National Public Health Laboratory

## References

[B1] Pandemic outbreak http://www.cnn.com/2009/HEALTH/06/11/swine.flu.who

[B2] Morbidity and Mortality Weekly Report. May 8, 2009 http://www.cdc.gov/mmwr/preview/mmwrhtml/mm5817a1.htm 19444147

[B3] Deadly new flu virus in US and Mexico may go pandemic New Scientist http://www.webcitation.org/5gNd9HTT9

[B4] Wiley DC Wilson IA Skehel JJ Structural identification of the antibody-binding sites of Hong Kong influenza haemagglutinin and their involvement in antigenic variation Nature 1981 289 373 8 10.1038/289373a0 6162101

[B5] Wilson IA Cox NJ Structural basis of immune recognition of influenza virus hemagglutinin Annu Rev Immunol 1990 8 737 71 10.1146/annurev.iy.08.040190.003513 2188678

[B6] WHO Swine flu pandemic has begun, 1st in 41 years 2009 The Associated press

[B7] Pandemic (H1N1) 2009 situation update-102, World Health Organization (WHO) http://www.who.int/csr/don/2009_09_11/en/index.html

[B8] 2009 H1N1 flu (Swine flu) and you http://www.cdc.gov/h1n1flu/qa.htm 19886299

[B9] Real-time PCR (Rrtpcr) for Detection and Characterization of Swine Influenza (Version 2009): Protocol. Center for Disease Control 2009

[B10] QIAmpR Viral RNA Mini Handbook Purification of Viral RNA (Spin Protocol) http://www.qiagen.com

[B11] New influenza A (H1N1) virus infections: global surveillance summary, May 2009 http://www.who.int/wer/2009/wer8420/en/index.html 19445090

[B12] Brownstein JS Wolfe CJ Mandl KD Empirical evidence for the effect of airline travel on inter-regional influenza spread in the United States PLoS Med 2006 3 1826 34 10.1371/journal.pmed.0030401 PMC1564183 16968115

[B13] Kwan-Gett TS Baer A Duchin JS Spring 2009 H1N1 influenza outbreak in king country Washington Disaster Med Pub Health Preparedness 2009 3 S109 S16 10.1097/DMP.0b013e3181c6b818 19952883

[B14] Mohammad AA Ziad AM Ali MA Pandemic Influenza A/H1N1 in Saudi Arabia: description of first one hundred cases Ann Saudi Med 2010 30 1 11 14 20103952 10.4103/0256-4947.59366 PMC2850176

[B15] Jain FS Finelli L Lindstrom S Garten RJ Gubareva L Bridges CB Emergence of a novel swine-origin Influenza A (H1N1) virus in humans N Engl J Med 2009 36 102 10.1056/NEJMoa0903810 19423869

[B16] Mathematical modelling of the pandemic H1N1 2009 Wkly Epidemiol Rec. Medline 2009 84 341 348 19702014

[B17] Wilson N Baker MG The emerging influenza pandemic: estimating the case fatality ratio Euro Surveill 2009 14 19255 19255 19573509

[B18] Fraser C Donnelly CA Cauchemez S Pandemic potential of a strain of influenza A (H1N1): early findings Science 2009 324 1557 1561 10.1126/science.1176062 19433588 PMC3735127

[B19] Presanis AM De Angelis D Hagy A The severity of pandemic H1N1 influenza in the United States, from April to July 2009: a Bayesian analysis PLoS Med 2009 6 e1000207 e1000207 10.1371/journal.pmed.1000207 19997612 PMC2784967

[B20] Donaldson LJ Rutter PD Ellis BM Mortality from pandemic A/H1N1 2009 influenza in England BMJ 2009 339 b5213 b5213 10.1136/bmj.b5213 20007665 PMC2791802

[B21] Writing Committee of the WHO Consultation on Clinical Aspects of Pandemic (H1N1) 2009 Influenza N Engl J Med 2010 362 1708 1719 10.1056/NEJMra1000449 20445182

